# The Significant Impact of High-Fat, Low-Carbohydrate Ketogenic Diet on Serum Lipid Profile and Atherosclerotic Cardiovascular Disease Risk in Overweight and Obese Adults

**DOI:** 10.7759/cureus.57920

**Published:** 2024-04-09

**Authors:** Shadan Khdher, Salm Mohammed, Kwestan Muhammed, Abdulrahman Ismael

**Affiliations:** 1 Clinical Biochemistry, Hawler Medical University, Erbil, IRQ; 2 Medical Laboratory Sciences, Cihan University, Erbil, IRQ

**Keywords:** overweight, obesity, lipid profile, ketogenic diet, cardiovascular risk, body mass index

## Abstract

Background and objectives

Overweight and obesity are becoming more commonplace globally. The ketogenic diet (KD), also known as the high-fat, low-carbohydrate diet, has become increasingly popular in recent years as a means to lose weight quickly. This present study aims to examine the clinical effects of ketogenic diets in individuals who are obese or overweight by evaluating or assessing variations in metabolic parameters associated with lipid control, the risk of atherosclerotic cardiovascular disease, and other kidney risk indicators.

Methods and subjects

This observational case-control research involved 250 individuals in total and was conducted from May 2023 to January 2024. Of these, 158 were on a ketogenic diet, and 92 adults not following any type of diet were chosen to serve as controls. The biochemistry parameters of the kidney function test and lipid profile were measured for the two comparing groups. Data were analyzed for statistical significance using the Student t-test, Mann-Whitney U test, and one-way analysis of variance (ANOVA), followed by a post hoc test (least significant difference (LSD)). Chi-square tests were employed in the analysis to compare proportions.

Results

Out of 250 participants, there was a 20-80 age range, with their median age being 40 years old. The two comparing groups' lipid profiles were very different from one another; the cardiovascular risk (triglyceride (TG)/high-density lipoprotein (HDL)), total cholesterol, low-density lipoprotein (LDL), and triglyceride levels were all greater in the KD group when compared to the non-KD group. The mean LDL cholesterol (LDL-C) of the normal-weight participants was 56 mg/dL (p=0.079). Thereafter, it experienced a significant rise to 97.58 mg/dL and 108.2 mg/dL in those individuals who were overweight and obese, respectively (p<0.020).

Conclusions

As obesity rates in the populace keep rising, dietary fads such as the ketogenic diet are gaining traction. Although they could help with weight loss, this study had a notable observation of severe hypercholesterolemia and increased risk of atherosclerotic cardiovascular disease among the ketogenic diet participants. Additional research is necessary to ascertain if a ketogenic diet can be sustained over the long term and how it affects endpoints that are more clinically significant, such as morbidity and mortality due to obesity.

## Introduction

Being overweight or obese results from gaining extra bodily fat that can harm human health. A variety of factors such as consuming too many calories, not exercising enough, hormonal imbalance, genetic factors, or a combination of these can lead to the accumulation of fat [1]. Using a body mass index (BMI) instead of a person's weight allows for a more precise estimation of body fat content. Body mass index (BMI), which is reported in kilograms per square meter, is computed by taking the person's weight in kilograms or pounds and dividing it by the square of their height in meters or feet [2]. Overweight is defined as having a BMI of 25 or higher, while obesity is defined as having a BMI of 30 or higher.

It is alarming to note that being overweight and obese is becoming more commonplace worldwide. In addition, its incidence has tripled since the 1970s, and the number of people who suffer from obesity is rising annually. It is estimated that by 2030, overweight and obesity will affect between 38% and 20% of the adult global population, respectively [3], and the American Medical Association (AMA) and the American Heart Association (AHA) are among the many medical societies who recognize obesity as a disease and one of the biggest health issues facing the world in the 21st century [4].

As obesity continues to become more prevalent and severe, it is becoming a significant underlying risk factor for chronic disorders such as cardiovascular diseases (CVDs), chronic renal disorders, malignancies, metabolic syndrome, and type 2 diabetes mellitus. In the long run, these conditions can lead to premature death and higher mortality rates in obese individuals [5].

Dietary restriction, more especially calorie restriction, is advised as the initial nutritional adjustment for weight loss in the current treatment guidelines for obesity. Lately, the ketogenic diet (KD) has gained a lot of popularity due to the studies that have been conducted showing its potential effectiveness in addressing weight gain [6]. A ketogenic diet consists of a high-fat, low-carbohydrate consumption that is restricted to 5%-10% of the daily required intake of carbohydrates and swapping the vast majority of it with nutritional fat and sufficient protein (1 g/kg) [7]. A typical KD often includes foods such as coconut oil, butter, cheese, avocado, eggs, and meat [8]. These foods are all high in fat and protein while low in carbohydrates, which is a cornerstone of a ketogenic diet.

The three main ketone bodies produced by mitochondria are acetone, acetoacetate, and 3β-hydroxybutyrate [9]. The breakdown of fatty acids into free fatty acids is the first step in the ketogenesis process. Following that, the free fatty acids are transferred from the adipocyte to hepatocyte, where they undergo oxidation to produce acetyl coenzyme A (acetyl CoA). Low-glucose conditions are where acetyl CoA is collected and converted by the enzyme thiolase into acetoacetyl CoA. Next, the production of β-hydroxy-β-methyl-glutaryl CoA (HMG CoA) from acetoacetyl CoA is catalyzed by the HMG CoA synthase. Then, acetoacetate and acetoacetyl CoA are produced from HMG CoA by HMG CoA lyase. Through non-enzymatic decarboxylation, acetoacetate can be further metabolized to acetone or 3β-hydroxybutyrate by β-hydroxybutyrate dehydrogenase [9].

To drive energy from ketone bodies, the body requires the process of ketolysis. This process involves the conversion of 3β-hydroxybutyrate and acetoacetate back into acetyl CoA through a two-step process that involves succinyl CoA: 3-oxoacid CoA transferase (SCOT) and acetyl CoA acetyltransferase (ACAT-1). Following its formation, acetyl COA enters the Krebs cycle and undergoes further oxidation, which produces adenosine triphosphate (ATP) molecules [10]. The results of a meta-analysis encompassing 15 research indicate that the very-low-calorie ketogenic diet leads to a notable reduction in body weight over the short, intermediate, and long term (-7.48 kg at one month, -16.76 kg at 4-6 months, and -21.48 kg at 12 months) [11].

Based on the results of every study that has been previously evaluated, the main goal of using a ketogenic diet for managing obesity is often to attain a notable decrease in total body weight and reduced fat mass content and BMI. However, it is unclear if the extremely low-calorie intake or the state of ketosis is to blame [12]. Short-term adherence to the ketogenic diet can cause mild side effects such as constipation, headaches, irritability, poor mode, and hunger [10], whereas long-term KD is associated with increased levels of circulating uric acid, an increased risk of kidney stones, and osteoporosis as a result of insufficient calcium ingestion [13].

However, there is still some debate over whether ketogenic diets are appropriate for obese people. As far as we are aware, no prior research on this topic has been conducted in Erbil. In light of this, the drive of this present study is to explore the clinical consequences of ketogenic diets in overweight or obese patients by estimating variations in metabolic parameters linked to lipid management, atherosclerotic cardiovascular disease (ACVD), and kidney risk indicators. The objective of this study is to investigate the relationship between lipid profile characteristics and the risks of cardiovascular disease and hypertension in individuals following a ketogenic diet.

## Materials and methods

Study design

This is an observational case-control study. The study's participants were divided into two distinct groups: group I (ketogenic diet), which encompassed 158 subjects on a ketogenic diet, who were strictly following a ketogenic diet and engaging daily (a 24-week ketogenic diet (consisting of 30 g carbohydrate, 1 g/kg body weight protein, 20% saturated fat, and 80% polyunsaturated and monounsaturated fat from total daily calories) [14] with different ages and gender) and group II (non-ketogenic diet), which encompassed 92 carefully chosen participants who were not following the ketogenic diet or any other diet employed to serve as controls.

Inclusion and exclusion criteria

Individuals 20 years of age and older of both sexes were enrolled. The following were the exclusion criteria: patients who had atherosclerotic coronary artery disease, suffering either from cancer or now receiving treatment for or having been found to have cancers, and suffered from beyond stage 3 chronic kidney disease, acute infectious diseases, concurrent autoimmune conditions, and chronic inflammatory diseases. They were also excluded from this study for better clarity of the laboratory test results. In the end, we also excluded patients who were not knowledgeable about the tests.

Blood sample collection

A total of 250 samples of blood were drawn from 158 subjects on the ketogenic diet and 92 subjects not on a ketogenic diet. As part of a medical procedure, the blood samples were collected in the morning after more than 12 hours of fasting, and a total of 5 mL of blood was drawn from each person using a syringe and needle for analysis. After the blood samples were collected, they were centrifuged at 3,500 rpm for 10 minutes. The separated serums used for the measurements included serum total cholesterol, serum high-density lipoprotein cholesterol (HDL-C), serum low-density lipoprotein cholesterol (LDL-C), serum triglyceride (TG), serum creatinine, blood urea nitrogen, and serum uric acid using COBAS Integra 400 plus biochemistry analyzer (Roche Diagnostics, Basel, Switzerland) by an enzymatic colorimetric method.

Study's timeline

The current study, which involved data collection, analysis, drafting, writing, and proofreading the manuscript, was conducted from May 5, 2023, to January 20, 2024, at Rzgary Hospital, Rozhhalat Emergency Hospital, and Surgical Specialty Hospital/Cardiac Center, Erbil, Iraq.

Statistical analysis

To perform the statistical analysis of the collected data, two software programs were used: GraphPad Prism version 9.5.1 (GraphPad Software Inc., San Diego, CA) and the Statistical Package for the Social Sciences (SPSS) version 25 (IBM SPSS Statistics, Armonk, NY). To determine whether the collected data was normally distributed, both the Shapiro-Wilk test and Kolmogorov-Smirnov test were used. The chi-square test of association was used to compare proportions. For categorical data, outcomes are presented as counts and percentages. A plot was made between the LDL and BMI categories using the GraphPad Prism software.

The Student t-test and Mann-Whitney U test for two independent sample groups (ketogenic and non-ketogenic groups) were used for the analysis. To compare three means by BMI categories, one-way analysis of variance (ANOVA) and the Kruskal-Wallis one-way ANOVA test were employed. After performing ANOVA, the means of each of the two groups were compared using a post hoc test (least significant difference (LSD)). The results are expressed as mean ± standard error of the mean for continuous variables. A p-value equal to or less than 0.05 was considered statistically significant, indicating that the observed differences between groups were unlikely to be due to chance.

Questionnaire form

In this study, a face-to-face, structured, direct interviewer-administered questionnaire was utilized as the data-gathering tool. Before its use in the study, the questionnaire was pretested and modified to ensure its reliability and validity. These participant's records were not readily available to the public and were used in the questionnaire to obtain a more comprehensive picture of the health status of the participants. The inquiry form covered demographic details including name, gender, address, and age. Additionally, the questionnaire also included questions about the family history, clinical risk issues, the patient's routine of smoking, and finally laboratory investigations such as serum lipid profile levels.

A calibrated digital scale was used to measure the study respondents' weight. Participants were asked to remove their shoes and wear loose clothes; it was estimated that the participants would lose 1 kg on average to make up for the weight of their clothes. The heights of the participants were expressed in cm utilizing a mobile measuring tape that was attached to the wall, and they were told to face the wall while standing barefoot. A person's weight in kilograms divided by their height in meters squared is their body mass index or BMI. According to the World Health Organization (WHO) guidelines, BMIs between 18.5 and 24.9 kg/m^2^ are considered normal in weight, 25 kg/m^2^ and above are regarded as overweight, and 30 kg/m^2^ and beyond are regarded as obese [15].

Ethical consideration

To conduct this study, the Hawler Medical University ethics committee granted ethical permission. Before they participated in the study, every participant received a thorough description of the purpose and context of the research. Clear oral permission was acquired from each participant after they were given an understanding of the data collection process and comforted about the privacy and authenticity of their data.

## Results

Two hundred fifty adults who visited Rzgary Hospital, Rozhhalat Emergency Hospital, and Surgical Specialty Hospital/Cardiac Center, Erbil, Iraq, between May 5, 2023, and January 24, 2024, were suitable for this study. Nevertheless, 10 KD patients and 21 control individuals decided not to participate, and in the end, 16 more cases were excluded due to additional types of diet. Of them, 63.2% were following KD, and 36.8% were not on KD. Their mean±standard deviation (SD) age in years was 40.85±14.90, the age range was 20-80 years, and the median was 40 years. The highest proportion of the sample (50.4%) was aged 20-40 years, and the gender distribution was nearly equal, with 50.4% being female and 49.6% being male. The largest part of the study population was overweight (about 45.6%), followed by obese individuals (about 41.6%), and only 32 (12.8%) were of normal weight. These characteristics and status are listed in Table 1.

**Table 1:** Baseline characteristics of the study participants BMI: body mass index, SD: standard deviation, COVID-19: coronavirus disease 2019, CVD: cardiovascular disease

Characteristics	Frequency	Proportions (%)/SD
Age groups		
20-40 years	126	50.4
41-60 years	110	44
61-80 years	14	5.6
Mean	40.85	14.90
Gender		
Male	124	49.6
Female	126	50.4
BMI		
Normal	32	12.8
Overweight	114	45.6
Obese	104	41.6
Previous COVID-19		
Yes	200	80
No	50	20
Smoking		
Smoker	176	70.4
Non-smoker	74	29.6
Family history of CVD		
Yes	80	32
No	170	68
Hypertension		
Yes	74	29.6
No	176	70.4
Diabetes mellitus		
Yes	138	55.2
No	112	44.8

Table 2 depicts the clinical characteristics of the KD group with a 24-week ketogenic diet and non-ketogenic diet individuals. The examination of the chi-square test revealed that there is a significant statistical discrepancy between the two comparing groups in age, hypertension, diabetes mellitus, previous COVID-19, and using hookah with a p-value of <0.001, <0.001, <0.001, <0.001, and <0.001, respectively.

**Table 2:** Clinical characteristics of the study groups based on the ketogenic diet COVID-19: coronavirus disease 2019

Characteristics	Ketogenic diet (number (%))	Non-ketogenic diet (number (%))	p-value
Hypertension	28 (17.7)	46 (50)	<0.001
Family history	46 (21.9)	34 (37)	0.200
Diabetes mellitus	62 (39.2)	76 (82.6)	<0.001
Smoking	110 (69.6)	66 (71.7)	0.723
Previous COVID-19	110 (69.6)	90 (97.8)	<0.001
Age (20-40)	106 (67.1)	20 (21.7)	<0.001
Age (41-60)	50 (31.6)	60 (65.2)	
Age (61-80)	2 (1.3)	12 (13)	
Hookah	124 (78.5)	52 (56.5)	<0.001
Gender (male)	84 (53.2)	40 (43.5)	0.140
Gender (female)	74 (46.8)	52 (56.5)	

Table 3 displays that there were notable differences in the lipid profiles of the groups. Expectedly, the KD group had a higher total cholesterol level (250.84±6.57) compared to the non-KD group (174.34±5.81) (p<0.001). The mean levels of HDL-C were significantly higher among the non-KD group (46.04±01.26) than the KD group (41.17±1.42) (p<0.001). The mean LDL-C values in the patients were higher (102.03±2.67) than in the control group (93.80±3.28), with highly significant p-value. The mean triglyceride levels were greater among the KD group (262.07±13.35) rather than in the non-KD group (222.80±18.43) (p<0.001). Participants from the KD group possessed a greater cardiovascular risk (TG/HDL) in comparison to those in the non-KD group (p=0.004). Following a 24-week KD, kidney function parameters were not significant.

**Table 3:** Metabolic parameters in individuals with KD and control non-KD *Data are analyzed using an independent t-test. **Data are analyzed using the Mann-Whitney U test. KD: ketogenic diet, SE: standard error, HDL-C: high-density lipoprotein cholesterol, LDL-C: low-density lipoprotein cholesterol, TG: triglyceride, BMI: body mass index

Parameters	Subjects	Mean±SE	p-value
Lipid profile			
Total cholesterol (mg/mL)*	KD	250.84±6.57	<0.001
	Non-KD	174.34±5.81	
HDL-C (mg/dL)**	KD	41.17±1.42	<0.001
	Non-KD	46.04±1.26	
LDL-C (mg/dL)**	KD	102.03±2.67	0.051
	Non-KD	93.80±3.28	
TG (mg/dL)*	KD	262.07±13.35	0.012
	Non-KD	222.80±18.43	
Cardiovascular risk (TG/HDL)**	KD	7.62±0.47	0.004
	Non-KD	5.44±0.56	
Kidney function test			
Blood urea nitrogen*	KD	39.04±1.02	0.369
	Non-KD	40.80±0.60	
Creatinine*	KD	1.41±0.61	0.510
	Non-KD	1.29±0.29	
Uric acid*	KD	8.62±0.12	0.596
	Non-KD	8.75±0.13	
Basic characteristics			
Age*	KD	34.58±0.96	<0.001
	Non-KD	51.61±1.36	
BMI*	KD	29.73±0.26	<0.001
	Non-KD	27.08±0.44	

It is evident in Table 4 that upon analyzing the different means of serum lipid profile by BMI categories among cases, a Kruskal-Wallis one-way ANOVA test showed that there are statistically significant differences between the four groups in all parameters. The mean LDL-C of the participants with normal weight was 56 mg/dL (p=0.079). Afterward, there was a significant rise to 97.58 mg/dL and 108.2 mg/dL in the cases of overweight and obese KD patients, respectively (p<0.020). It is obvious that the subjects with normal weight had an average total cholesterol of 87 mg/dL; thereafter, the levels experienced a significant rise to 251.78 mg/dL in the case of obese individuals and 254 mg/dL in the case of overweight individuals (p=0.017). The results were similar for triglycerides.

**Table 4:** Means of serum lipid profile by BMI categories among ketogenic diet subjects *One-way ANOVA test **Kruskal-Wallis one-way ANOVA test BMI: body mass index, ANOVA: analysis of variance, SE: standard error, LSD: least significant difference, HDL-C: high-density lipoprotein cholesterol, LDL-C: low-density lipoprotein cholesterol

Parameters	BMI (kg/m^2^) groups	Means±SE	p by ANOVA	LSD groups	p (LSD)
Total cholesterol (mg/mL)*	Normal (A)	87.0±0.00	0.017	A X B	0.005
	Overweight (B)	254.0±65.39		A X C	0.005
	Obese (C)	251.78±11.14		B X C	0.864
HDL-C (mg/dL)**	Normal (A)	45.00±0.00	0.054	A X B	0.574
	Overweight (B)	37.87±0.99		A X C	0.984
	Obese (C)	44.73±2.78		B X C	0.017
LDL-C (mg/dl)**	Normal (A)	56.00±0.00	0.020	A X B	0.079
	Overweight (B)	97.58±3.24		A X C	0.028
	Obese (C)	108.21±4.25		B X C	0.046
Triglycerides (mg/dL)*	Normal (A)	73.00±0.00	0.028	A X B	0.066
	Overweight (B)	291.64±20.38		A X C	0.174
	Obese (C)	234.41±16.47		B X C	0.032

**Figure 1:**
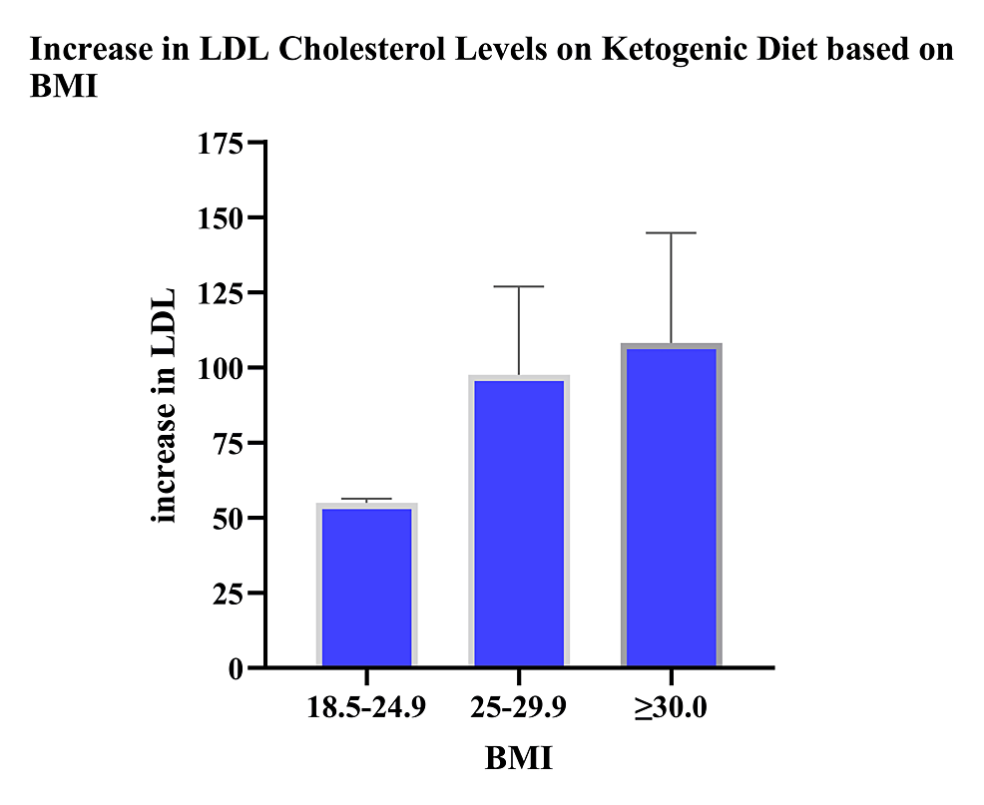
Impact of ketogenic diet on LDL cholesterol levels based on BMI levels LDL: low-density lipoprotein, BMI: body mass index

Interestingly, we saw the largest percent increase in LDL cholesterol levels in patients with higher BMI (Figure 1). LDL cholesterol levels increased significantly in tandem with the rise of BMI.

It is clear in Table 5 that the percentage of patients with hypertension rose dramatically (p<0.007) from 0% of individuals who were normal weight to 26.8% of those who were overweight. Additionally, the percentage of individuals with diabetes mellitus rose from 0% among those who were normal weight to 43.2% among those who were obese, although statistically not meaningful.

**Table 5:** Distribution of clinical characteristics by BMI categories among ketogenic diet individuals BMI: body mass index, COVID-19: coronavirus disease 2019

KD subjects (N=158)				
Categorical variables	Normal (18.5-24.9 kg/m^2^) (A) (n=2)	Overweight (25-29.9 kg/m^2^) (B) (n=82)	Obese (≥30 kg/m^2^) (C) (n=74)	p-value
Gender, male (number (%))	0 (0)	44 (53.7)	40 (54.1)	0.316
Gender, female (number (%))	2 (100)	38 (46.3)	34 (45.9)	0.316
Diabetes mellitus (number (%))	0 (0)	30 (36.6)	32 (43.2)	0.361
Hypertension (number (%))	0 (0)	22 (26.8)	6 (8.1)	0.007
Previous COVID-19 (number (%))	2 (100)	62 (75.6)	46 (62.2)	0.122
Family history (number (%))	0 (0)	24 (29.3)	22 (29.7)	0.658
Smoking (number (%))	0 (0)	64 (78)	46 (62.2)	0.010

## Discussion

Like across Iraq, the frequency of overweight and obesity is rising in the Kurdistan area and is becoming a serious health concern [15]. Obesity is a significant risk factor for many long-term diseases, including diabetes mellitus, hypertension, heart attack, and other cardiovascular issues. Several pathophysiological processes have been proposed to link obesity and cardiovascular illnesses. One such mechanism is hypercholesterolemia, which is currently thought to be the primary factor in the development of atherosclerotic coronary artery disease [16]. Therefore, it is impossible to overstate the importance of a healthy regimen in the control and avoidance of chronic illnesses, and there are already many dietary programs provided to assist in weight loss or eliminate complications caused by obesity, including a traditional diet [17].

This investigation assessed the lipid profile values of patients following the high-fat, low-carbohydrate "ketogenic diet." In contrast to the group under control, we discovered that patients who followed the KD for 24 weeks on average had a considerably higher mean of total cholesterol and LDL-C (p<0.001 and p=0.051). The significant increase in LDL-C values that we saw in our patients on the KD might be attributed to a number of factors. One theory is that the diet involves increased consumption of meat, particularly red meats such as beef, which is directly linked to elevated lipid levels. This outcome is consistent with several earlier investigations conducted by Kirkpatrick et al. [18] and Bueno et al. [19].

Furthermore, we examined a ketogenic diet's impact on cardiovascular risk indicators and lipid controls in the current study and discovered that both overweight and obesity were linked to a heightened risk of cardiovascular illnesses, including hypertension. Additionally, every patient with KD thereafter tends to have low HDL are two distinct risk factors for insulin resistance and cardiovascular diseases. Other studies [20,21] that assessed the results of a diet low in carbohydrates found comparable outcomes.

Our present study reduced the likelihood of any potential causes for increasing or decreasing laboratory levels by using healthy individuals to serve as controls who had no prior history of illnesses or ailments that may have an impact on the accuracy of the results. Nonetheless, the investigation by Choi et al. [5] found that the ketogenic diet's impact on kidney function tests was equivalent to that of the control group. These outcomes are consistent with what we investigated. In a similar vein, Rosemary and Bibiana [22] discovered that their research's findings did not significantly change the renal parameters of rats who had been given KD, supporting the conclusion that a high-fat KD does not cause harm to the kidney.

It is noteworthy to notice that individuals on the ketogenic diet who had greater body mass indices also had higher percentage increases in LDL cholesterol. The proportion of clinically elevated LDL-C values rose with increasing BMI, reaching 97.58% in overweight persons and 108.21% in obese individuals. Schmidt et al. [22] observed that KD patients with greater BMI had lower LDL cholesterol levels, but their study lacked a control group for comparison, and it was a case-based report, therefore weakening the association.

Several factors affect the correlation between BMI and LDL levels in individuals following a ketogenic diet. Primarily, a ketogenic diet typically involves a high-fat, low-carbohydrate intake that leads to alterations in metabolism and energy usage. These modifications are a result of the increased consumption of dietary fats and the body's dependence on fat as a source of energy. Moreover, the genetic factors, specific dietary choices within the ketogenic approach, and the duration of the diet are all factors that contribute to the variations while evaluating the relationship between BMI and LDL levels in individuals on a ketogenic diet. Neglecting these factors can lead to variations and put our overweight and obese patients at a significantly increased risk of cardiovascular disease.

According to the findings of this study, consuming a lot of meat and other animal products, such as tallow, may raise your triglyceride and cholesterol levels. One possible tactic to lessen or mitigate the adverse impacts of KD on LDL-C is to substitute polyunsaturated fats for animal-based saturated fats, which you may get from foods such as avocados, almonds, coconut oil seeds, and olive oil. Based on this and in light of the study's findings, we recommend that all essential steps be taken to modify diet, exercise routine, and way of life to keep BMI within normal bounds and thereby lower the incidence of chronic illnesses such as cardiovascular disease.

Strengths and limitations

In light of what we know, this is the first study carried out in Erbil to evaluate perceptions around the ketogenic diet and its impact on lipid metabolism. The sample size that was gathered was sufficient for the objectives of this investigation. As much uniformity as possible was maintained throughout the interviews, and when participants were questioned about their diets, questions were asked in an open-ended fashion with little to no direction. However, because the individuals were patients attending a public hospital or healthcare center, they were likely more health-concerned than the general population, raising the possibility of sampling bias. The results of the study might not be representative of the entire inhabitants in this region because it was limited to three healthcare facilities in a single city. Since the data points were self-reported, there is also a chance of recollection bias.

## Conclusions

This study revealed severe hypercholesterolemia and an increased risk of atherosclerotic cardiovascular disease among the majority of individuals who were following a ketogenic diet. Its effects on other risk markers and kidneys were comparable to those of the control group. It seems that the ketogenic diet is not safe to be used for more than six months. Instead, it should be used with caution and be closely monitored by periodic laboratory testing of blood ketones, lipids, and other relevant biochemical indicators including kidney and liver function tests. More investigation is required to determine if a ketogenic diet is sustainable in the long run and how it impacts endpoints that are more clinically meaningful, such as obesity-related morbidity and death.
